# Ontogeny of foraging behaviour in juvenile red-footed boobies (*Sula sula*)

**DOI:** 10.1038/s41598-017-14478-7

**Published:** 2017-10-24

**Authors:** Loriane Mendez, Aurélien Prudor, Henri Weimerskirch

**Affiliations:** Centre d’Etudes Biologiques de Chizé (CEBC), UMR7372 CNRS, Université de La Rochelle, 79360 Villiers-en-Bois, France

## Abstract

The early life stages represent a crucial period that can strongly influence population dynamics. We studied the development of foraging behaviour in the red-footed booby, a tropical seabird with an extensive post-fledging care period (3 to 6 months). Adults and juveniles were observed from shore and tracked at sea using GPS loggers over 3 consecutive 12-day periods. Juveniles initially made a majority of flights inland, likely to practice flying, and formed groups of up to 10 juveniles before making short trips at sea. They left the island later and returned earlier than the adults, allowing them to be fed on the nest. Over time, juveniles left the colony alone more frequently and increased the range of their trips while remaining significantly closer to the colony than the adults. They spent more time intensively foraging (slow and sinuous trajectory) than adults, which could reflect attempts to capture prey. Juveniles foraged independently of their parents but associated frequently with congeners, particularly during area-restricted search (ARS) behaviour. The extensive post-fledging care period observed may be explained by the need to develop proper foraging skills adapted to tropical waters, where resources are particularly scarce and unpredictable.

## Introduction

The early life stages usually represent a critical period in the life-history of animals^1^. Young individuals often experience substantial mortality, directly impacting population dynamics^2,3^. This high mortality is generally assumed to be the result of the difficulty of young individuals to find and acquire sufficient food for survival^4^. Foraging efficiency is thought to be improved through learning and foraging experience but is also believed to be constrained by physical development^5^. However, little information is available on the development of foraging skills during the juvenile phase. In particular, few studies have focused on how juveniles use social information from the behaviour of congeners to make foraging decisions^6^.

The influence of conspecifics on foraging behaviour in vertebrates has been extensively studied for decades^7^. Individuals can unintentionally provide information through cues and signs that congeners or other species can detect and exploit. For example, some fish species will exploit public information if their personal information about a food patch is unreliable or outdated^8^, or will use the same route as other individuals even if it is longer and more energetically costly than alternative routes^9,10^. By observing the foraging success of other individuals to modify their own foraging behaviour^11,12^, certain assemblages of birds are used as ‘information centres’ for finding food^13^. More recently, miniaturised video recorders have provided clear evidence of frequent at-sea associations in structured networks among foraging Cape gannets (*Morus capensis*)^14^. During the juvenile stages, social learning may be an important strategy to gain foraging skills and experience. Influential sources of information can be provided by related conspecifics, as in juvenile Florida scrub-jays (*Aphelocoma coerulescens*) that can forage in a novel patch when they are in proximity to other family members that foraged successfully in that patch^15^. Signs and clues can also be provided by unrelated conspecifics, as in juvenile ringdoves (*Streptopelia risoria*) that can acquire social information from whatever knowledgeable individuals they observe^16^. The foraging behaviour of juvenile seabirds has been investigated only recently^17–26^ and is therefore not fully understood. It is not known, for example, whether juvenile seabirds learn on their own or join groups of adult or immature congeners to mimic their foraging behaviour.

In seabirds, juveniles generally leave their birth colony for the open ocean during their first flight^27^. Fledglings can disperse over vast areas^19^ and must learn foraging skills without assistance from their parents^27^. High mortality is generally observed during the first months after the fledglings leave the nest for the first time; this is presumably related to the foraging failure of naive birds^8,18,22,23,28^. The few direct observations that exist for foraging juveniles suggest that young seabirds foraging nearshore encounter difficulties in locating food sources with lower attempt and/or success rates compared to adults^27^. In some seabird species such as boobies or frigatebirds, juveniles pass through a transition phase whereby they practice flight and leave the colony to forage at sea, but return to the nest to be fed by their parents^27^. This transition phase can last several months^29–31^ and is thought to improve flight capabilities^32^ and allow the development of hunting techniques and the efficient search for favourable foraging areas^17,25^.

The red-footed booby (*Sula sula*), hereafter RFB, is the most pelagic of the boobies^29,33^. The species is widely distributed throughout the pantropical latitudes in the Atlantic, Pacific and Indian Oceans^29^. The female lays one egg and the two partners of a pair take turns at sea to forage and to feed the chick after hatching. The point at which the young leaves the nest for the first time is called fledging and occurs approximately 130 days after hatching^34^. After fledging, the young returns to its nest every night to be fed regularly by its parents^20^. The duration of the post-fledging period, defined as the period between the first flight from the nest and the time when the young is completely independent of its parents, lasts between 90 and 180 days^20,34,35^.

We studied the development of the foraging behaviour of juvenile RFBs during the transition phase, *i.e*., from their first flight over land until they forage regularly at sea. During 3 consecutive 12-day periods (hereafter referred to as P1, P2, P3), both adults and juveniles from the breeding colony on Europa Island (Mozambique Channel) were tracked with GPS loggers. Additional visual surveys of their social behaviour and movements were carried out from the island. The aim of the study was to compare the behaviour of juveniles with that of adults and to investigate the changes in behaviour occurring over the transition phase. We expected behavioural changes over time in juveniles, unlike adults, which should undertake optimised foraging trips to feed both their young and themselves. We predicted that juveniles would increase their foraging effort over time, whereas adults would progressively decrease provisioning. Our tracking information, combined with visual observations of groups during departure and return, allowed us to test for the first time whether some form of association between juveniles and adults occurred during this transition phase. Visual surveys and camera traps were used to monitor the nests and better understand how and when the juveniles became independent.

## Results

### Departure and return from the colony

The behaviour of young fledglings was investigated after their first flight during 3 consecutive periods of 12 days (P1, P2 and P3). Throughout the entire study period, the juveniles tracked by GPS made short flights over the island (7.2 ± 7.6 min) and longer trips over the sea (4.8 ± 2.6 h). The proportion of flights over the sea progressively increased from 20% for period 1 (P1) to 30% for period 2 (P2) and 45% for period 3 (P3).

**Figure 1:**
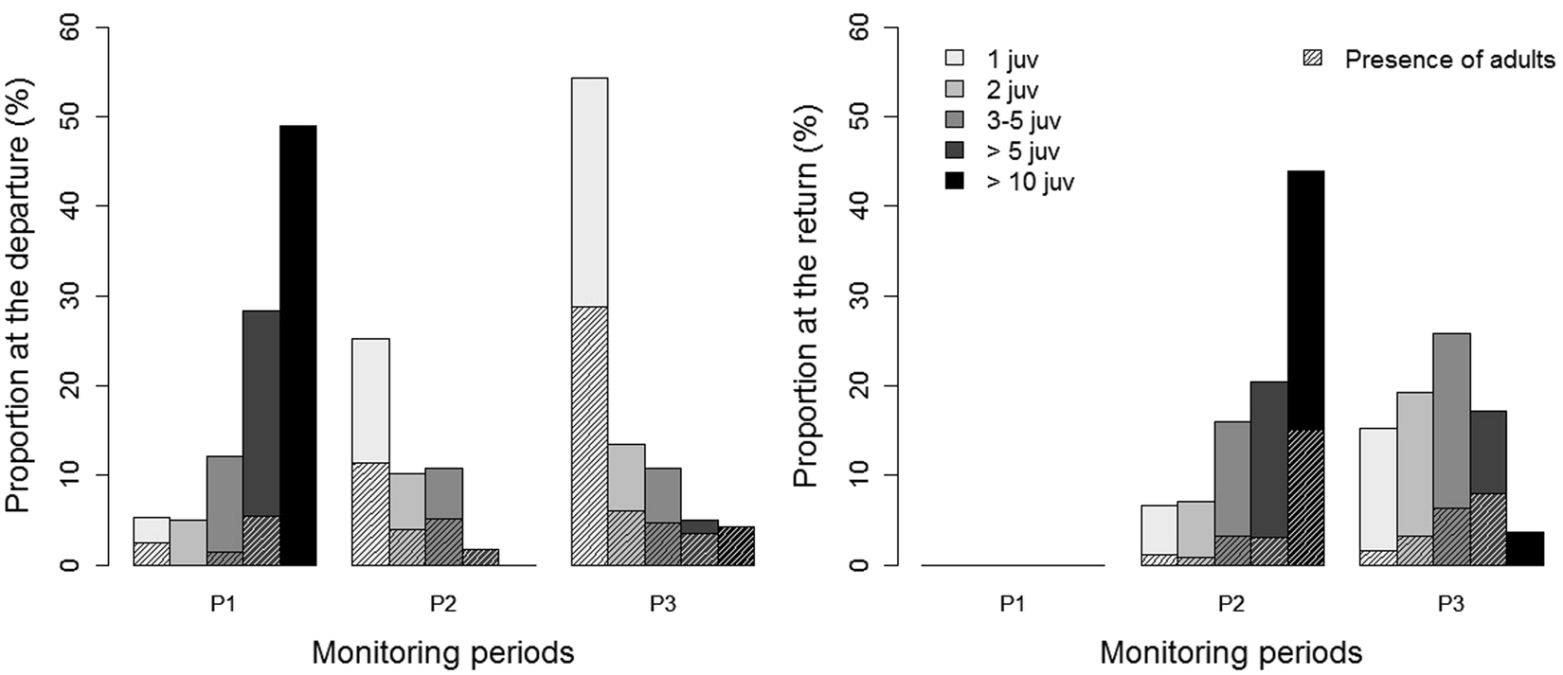
Proportion (%) of juvenile red-footed boobies departing from (left panel) and returning to (right panel) the colony alone (1 juv.), in pairs (2 juv.), in groups of 3 to 5 juveniles (3–5 juv.), in groups of more than 5 juveniles (>5 juv.) and in groups of more than 10 juveniles (>10 juv.) during the 3 consecutive monitoring periods (P1, P2 and P3). Hatching indicates the simultaneous presence of adults. No monitoring of returns was conducted during P1.

Visual surveys from the colony revealed that juveniles headed to sea and returned to land alone or in groups of variable size and composition. The distribution of the different group formations during departure was significantly different among the 3 periods (χ² = 492.58, df = 8, p < 0.001; Fig. 1). During P1, the majority of juveniles left the colony in large groups composed of more than 10 of juveniles (up to 55 individuals). Over time, a higher proportion of juveniles left the colony alone rather than in a group. The distribution of group formations between the departure and the return was significantly different for P2 (χ² = 250.48, df = 4, p < 0.001) and P3 (χ² = 121.96, df = 4, p < 0.001). In fact, unlike their departure, juveniles returned primarily in groups during P2 and P3, suggesting that groups were formed at sea. Upon return, the distribution of the group formations was significantly different between P2 and P3, with fewer observations of large groups over time (χ² = 153.52, df = 4, p < 0.001). No monitoring of returns to the colony was made during P1.

During P1, juveniles left the colony accompanied by adults in less than 10% of the observations (Fig. 1). During P2 and P3, they joined groups with adults in approximately 50% of the cases when leaving the colony. Upon return from the sea, juveniles returned to the colony with adults in 25% of the cases, regardless of the period.

### Characteristics of foraging trips

**Figure 2:**
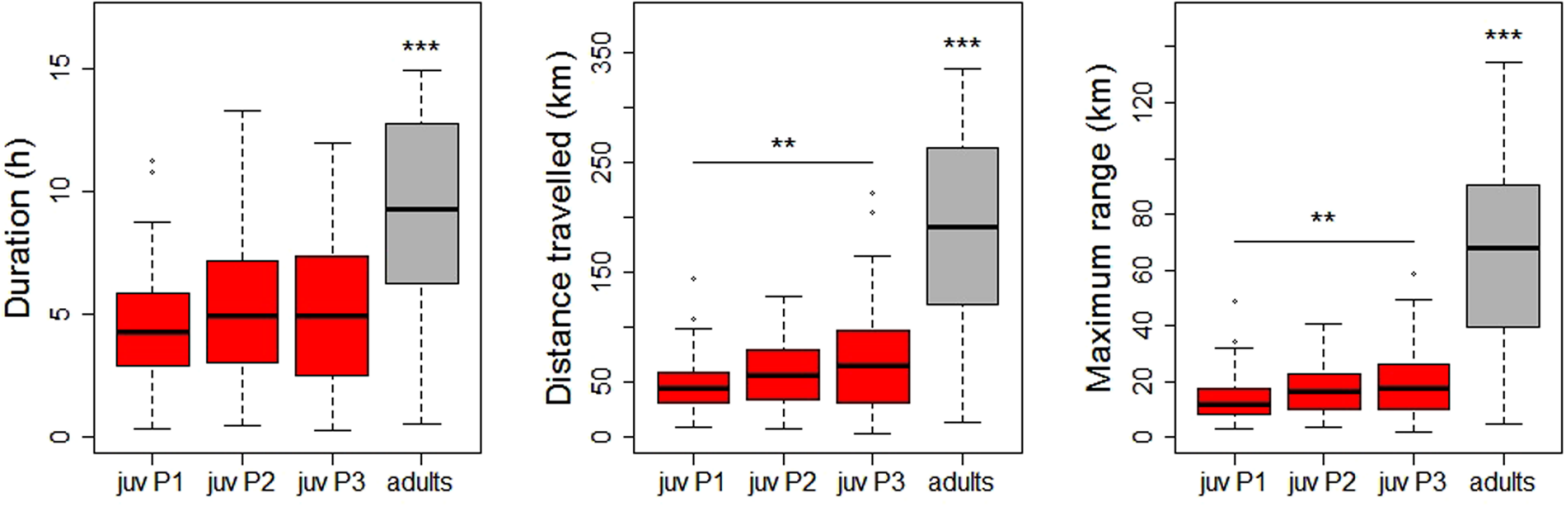
Duration (h), distance travelled (km) and maximum range (km) of the foraging trips of juvenile (red) and adult (grey) red-footed boobies during the 3 consecutive monitoring periods (P1, P2 and P3).

Both adults and juveniles typically performed one trip at sea per day. Days including 2 trips by the same individual represented 3.6% and 5.5%, respectively, of the total tracking days for juveniles and adults. Trips at sea lasted from 0.3 to 14.9 hours (Fig. 2). Juveniles (all periods combined) made significantly shorter trips than adults (4.8 ± 2.6 h and 9.1 ± 4.0 h, respectively; Tukey’s HSD test, p < 0.001). The duration of trips by juveniles was not significantly different among the 3 periods. The distance travelled by juveniles increased significantly over the 3 periods (from 47 km to 68 km; Tukey’s HSD test, p = 0.002), and remained significantly shorter than the adults’ trips (178 km ± 83 km; Tukey’s HSD test, p < 0.001). The same pattern was observed for the maximum range of juveniles as the distance travelled increased from an average of 14 km to 19 km over time (Tukey’s HSD test, p = 0.009); juveniles remained significantly closer to the colony than adults (65 km ± 33 km; Tukey’s HSD test, p < 0.001).

Six juveniles were tracked during 13 to 29 consecutive days over the 3 periods (see Supplementary Fig. S1). For 5 of these individuals, the maximum range (distance from the colony) significantly increased over time (Pearson’s correlation coefficient 0.47 ≤ r ≤ 0.61, 0.002 ≤ p ≤ 0.05). These 5 individuals travelled more than two times further between their first day and the last day of tracking.

Departure and return times were evaluated for all tracks (see Supplementary Fig. S2). Juveniles departed progressively earlier throughout the 3 successive periods (12:00, 11:00 and 10:00 on average), but still departed significantly later than the adults (09:00 on average; Tukey’s HSD test, p < 0.001, p < 0.001 and p = 0.012, respectively). Throughout the monitoring period, juveniles typically returned to the colony between 15:00 and 16:00, significantly before the return of the adults (18:30 on average; Tukey and Kramer test, p < 0.001 for the 3 pairwise comparisons).

### Behaviour clustering and area-restricted search

**Figure 3:**
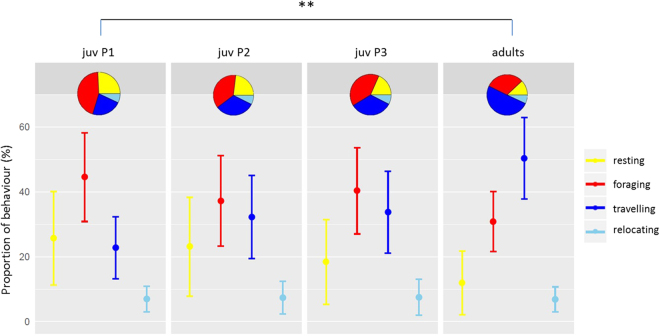
Proportion (mean ± SD) of behaviours (resting, intensive foraging, travelling, relocating) assigned along the tracks of juveniles during the 3 consecutive monitoring periods (P1, P2 and P3) and along the tracks of adult red-footed boobies.

We used the Expectation Maximisation binary Clustering (EMbC) algorithm to characterise and compare the behaviour of adults and juveniles (Fig. 3). Depending on the speed and turning angle, each location could be labelled resting (slow and straight trajectory), intensive foraging (slow and sinuous trajectory), travelling (fast and straight trajectory) or relocating (fast and sinuous trajectory). The latter may reflects a reorientation between different patches of prey in a globally favourable zone. The distribution of the different behaviours among the foraging trips at sea was significantly different between juveniles and adults during P1 (χ² = 19.56, df = 1, p = 0.025). Juveniles were more frequently engaged in intensive foraging and less frequently engaged in travelling compared with the adults. The proportion of time spent resting and relocating were similar for adults and juveniles. During the first period, juveniles spent a large proportion of time intensively foraging during their trips (45% on average). This proportion decreased during P2 and P3 (37% and 40%, respectively). Juveniles exhibited a similar distribution of behaviours during P2 and P3. Conversely, adults spent more time travelling, particularly when compared with juveniles during P1.

Area-restricted search (ARS) zones were defined as a succession of locations identified as “intensive foraging” by the EMbC algorithm. The ARS zones of juveniles and adults partially overlapped around the island, but the adults used a total area approximately 5 times larger than that of juveniles (see Supplementary Fig. S3). The surface of the core (50% kernel) and general (95% kernel) areas of intensive foraging were 378 km² and 2 190 km², respectively, for the juveniles and 7 048 km² and 31 784 km², respectively, for the adults.

During P1, juveniles performed significantly more ARSs per hour than the adults (0.99 ± 0.33 h and 0.82 ± 0.31 h, respectively; Tukey’s HSD test, p = 0.010) but the ARS zones showed similar patterns for juveniles and adults (see Supplementary Fig. S4). The duration of the ARS was highly variable with similar ranges for juveniles and adults (median value 21 min). The mean surface of the ARS was also similar for juveniles and adults (1.35 km² ± 2.4 km², not shown in Fig. S4). The distance of the ARS zones from the colony increased slightly across the 3 monitoring periods for juvenile birds with significantly different values between P1 and P3 (from 12.46 ± 7.5 km to 17.70 ± 10.38 km, respectively; Tukey’s HSD test, p = 0.005). The ARS locations of adults were much farther from the colony (62.90 ± 32.80 km) compared with the 3 successive monitoring periods of juveniles (12.5 ± 7.5 km, 15.4 ± 8.1 km, 17.7 ± 10.4 km; Tukey’s HSD test, p < 0.001 for the 3 pairwise comparisons).

### At-sea associations

**Figure 4:**
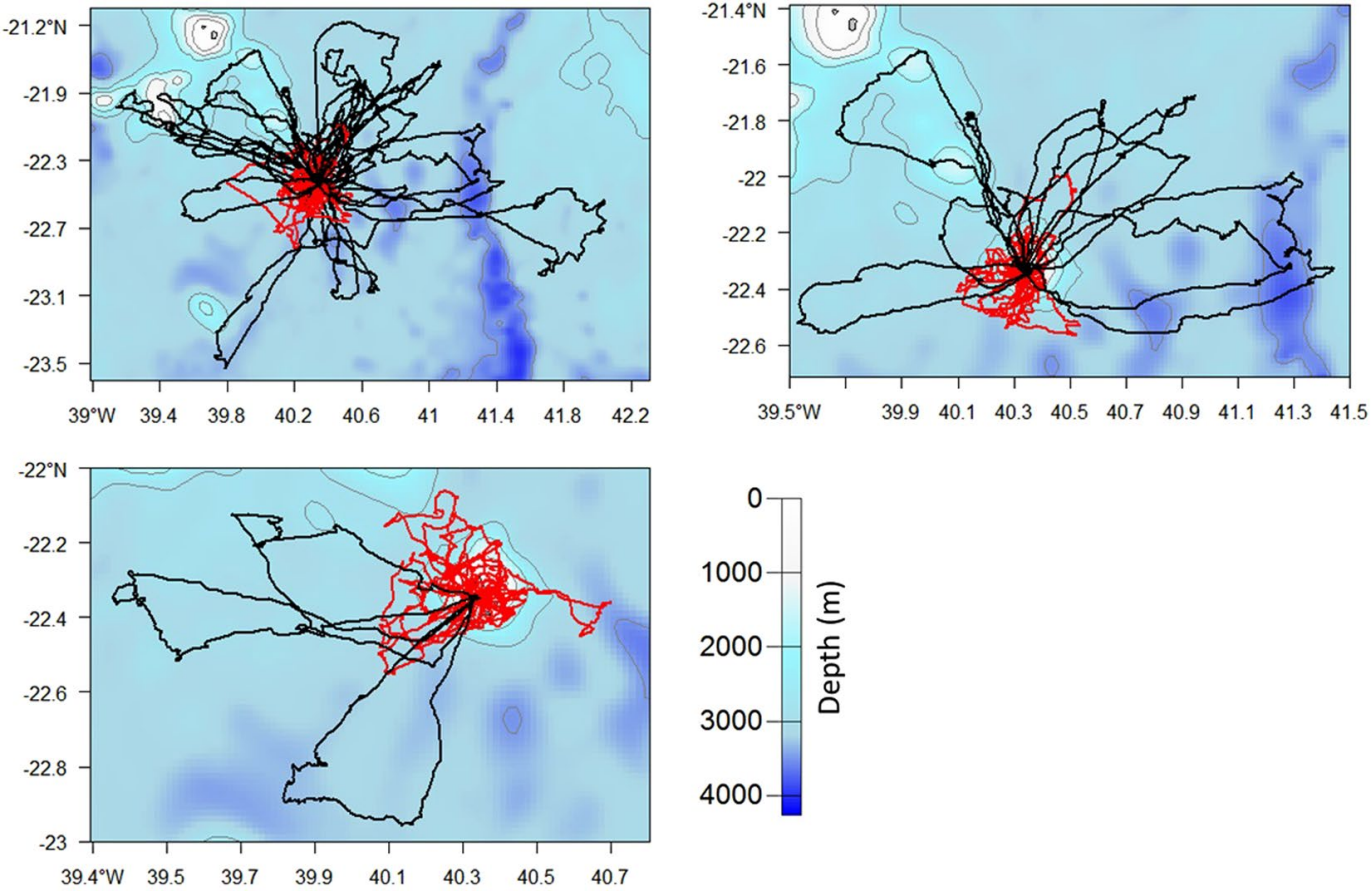
Foraging trips of 3 different pairs of a parent red-footed booby (black) and its juvenile (red). Tracks are superimposed on bathymetric maps generated with the R package *marmap* using the software R version 3.2.3 (2015-12-10, www.R-project.org).

No association was observed between the foraging trips of one parent tracked simultaneously with its own juvenile in the 3 study cases (Fig. 4). Each successive trip was oriented in variable directions from one trip to the next, and the juvenile did not follow the parent’s trajectory.

**Figure 5:**
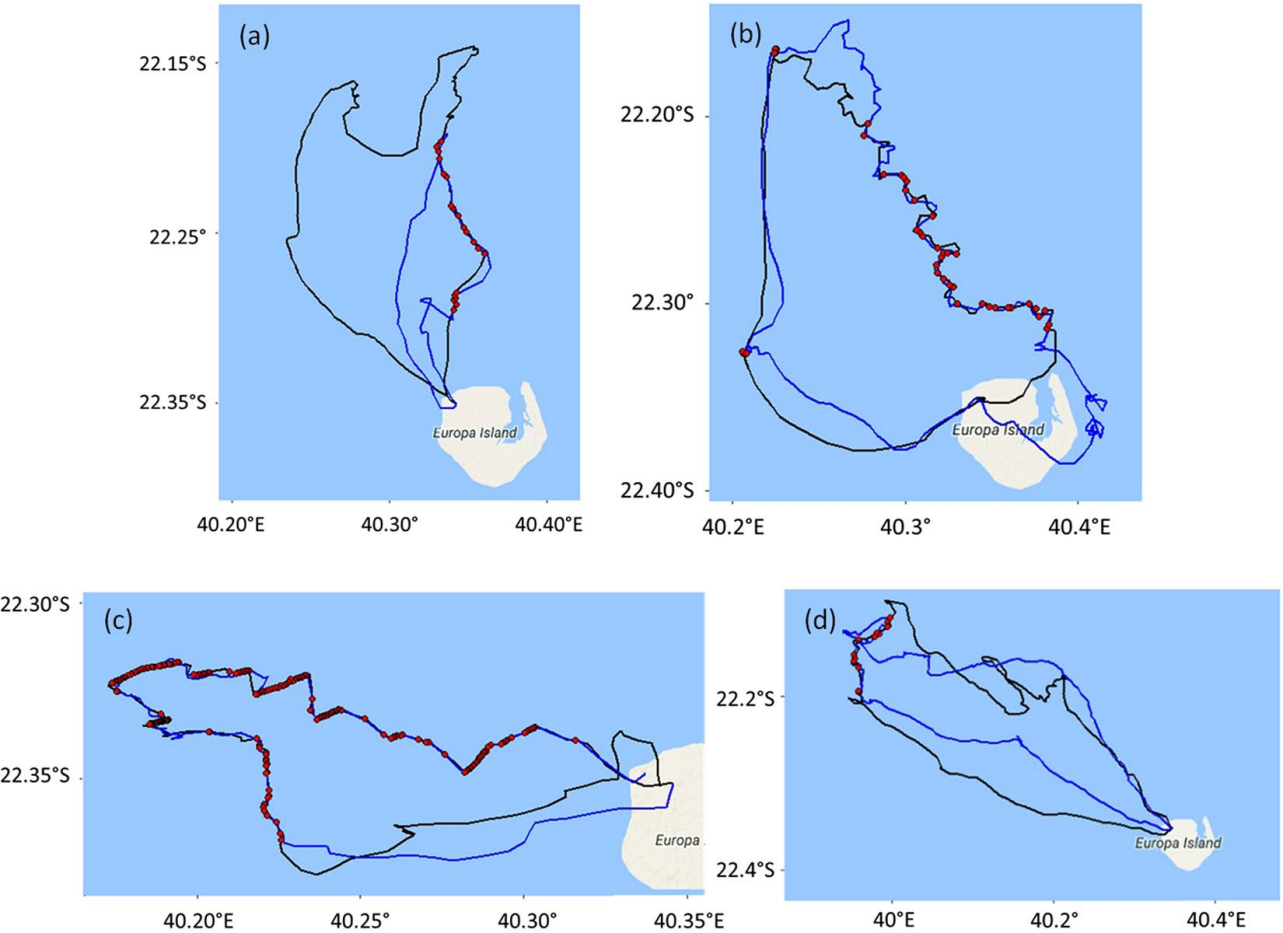
(**a**–**c**) Examples of associations (red) between 3 different pairs of juvenile red-footed booby (blue and black); (**d**) Example of an adult red-footed booby and an unrelated juvenile that accompanied the adult for a short foraging period. Associations between 2 locations were identified as the differences in latitude and longitude < 0.002 within a time interval of 30 s. Tracks are superimposed on maps generated with the R package *ggmap* (map data ©2017 Google) using the software R version 3.2.3 (2015-12-10, www.R-project.org).

Associations between tracked juveniles (Fig. 5a–c) occurred on 54% of the tracks (171 associated tracks on 316 tracks in total). Associations between a juvenile and an unrelated adult were only observed on 2 tracks (of 316 tracks in total), including one track where they foraged together during the middle of their respective trips (Fig. 5d). When associations occurred along a track, birds were associated with one or two other birds in 70% of all cases. Birds were associated with 3 other birds in less than 15% of all cases, but up to 9 birds were found to be simultaneously associated at some stages of the same foraging trip. The durations of the associations among juveniles were highly variable depending on the tracks and represented 14% ± 13% of the duration of the track. Associated parts of the track represented less than 5% of the duration of the track in more than 60% of the tracks. The proportion of associated locations increased with the number of birds associated during the track.

We tested whether the associations between juveniles were more likely to occur during ARS behaviours. ARS behaviours represented 39% of all juvenile GPS locations. Under the null hypothesis that ARS behaviour and pair associations were independent, we could expect to find 37% of associations with no individual in ARS behaviour (p_0_ = 0.61*0.61), 15% of associations (p_2_ = 0.39*0.39) with 2 individuals in ARS and 48% with only one individual in ARS (p_1_ = 1 – (p_0_ + p_2_)). We found that p_0_ = 27%, p_1_ = 29% and p_2_ = 44%, indicating that associations were more likely to occur during ARS behaviour (χ² = 1568.9, df = 2, p < 0.001).

### Feeding frequency and juvenile independence

Shortly after fledging, all juveniles returned to the nest each day. The juvenile was fed by its parents 1 to 3 times a day, with an average frequency of 1.38 feeding events per day (Table 1) during the first two weeks after fledging. During that period, the juveniles were slightly smaller than the adults (see Supplementary Fig. S5). Tracked juveniles had a similar wing length (F = 0.1645, p = 0.69) but a significantly shorter beak than adults (culmen length: F = 11.792, p < 0.001; culmen height: F = 34.392, p < 0.001). Approximately 60 days after fledging, only half of the juveniles were present on the nest at night, and the feeding frequency was close to one feeding event per day. Twenty days later, only 20% of the juveniles were still present on the nest at night, and the feeding frequency was only 0.10 feed per day (Table 1). Finally, no juveniles were observed on the nest during an opportunistic observation 155 days after fledging (Table 1).

**Table 1:** Presence of juveniles on the nest at night and the feeding frequency by adults from camera trapping. n_days_ = number of days of surveys.

Period after fledging	Presence on nests (at night)	Feeding frequency (day^−1^)
+15 days	100%	1.38 (n_days_ = 13)
+60 days	50%	1.06 (n_days_ = 15)
+80 days	19%	0.10 (n_days_ = 20)
+155 days	0%	—

## Discussion

The simultaneous use of GPS tracking, visual surveys and camera trapping provided valuable information on changes in social and foraging behaviour of a seabird during the early stage of independence. This transitional phase appears to be a phase of learning that allows young individuals to acquire foraging skills. RFBs feed primarily on flying fishes and flying squids that are caught in flight when these prey emerge from the surface or are caught just below the surface after a short plunge dive^36^. These foraging skills are probably complex and difficult to acquire, requiring several weeks or months of practice. In addition, tropical seabirds generally feed in association with sub-surface predators such as tuna and dolphins, which drive prey close to the ocean’s surface^37,38^. Because the location of these prey patches is considered to be unpredictable^39^, learning how to detect these feeding opportunities probably requires additional time. All these constraints may explain the particularly long transition phase observed in boobies and frigatebirds^17,27,32,40^ that have a similar diet^41,42^.

Underdeveloped flying skills may be one of the proximate causes of the long post-fledging care period observed in some seabird species^32,43^. During the entire monitoring period, a large proportion of flights by juvenile RFBs were short, exploring only the interior of the island; this was particularly true during the first monitoring period, when they were probably still acquiring flight skills. Young juveniles formed groups along the coast before going to sea and tended to return in small groups and rarely returned alone. During the subsequent periods, the observation that birds were returning in larger groups than when they left the island indicated that some associations were formed at sea, in addition to those formed at departure. This was confirmed by the simultaneous tracking of several juveniles. All birds, juveniles as well as adults, returned to the colony before nightfall on the same day that they left the island. During the post-fledging period, juvenile RFBs are still fed by their parents and thus probably experience little energetic pressure to forage for their own needs. Our observations were consistent with those of Guo *et al*.^20^, who noted that juvenile RFBs left the island later in the day than the adults and returned earlier as they needed to be on the nest before their parents’ return. Throughout the entire visual monitoring period, juveniles were rarely observed returning to the colony with adults, probably because adults return to the colony later. Indeed, adults are under severe energetic pressure to make foraging trips that are long enough to bring food for their young and to feed themselves. The trips of adult RFBs were typically highly directed, leaving and returning to the colony with a straight trajectory. Their primary foraging activity was concentrated in the middle of the track (in terms of duration), often at the furthest point of the trip from the colony^42^.

Compared with adults, juveniles did not travel as far from the colony, potentially because of undeveloped flying or foraging skills and the need to return to the colony before the parents to be fed. The slight difference in size between the adults and the juveniles did not appear to be an important determinant of the difference in range. Similar to juvenile wandering albatrosses (*Diomedea exulans*) and immature common guillemots (*Uria aalge)*, the foraging areas of juvenile RFBs showed little overlap with those of adults^44,45^. This habitat partitioning could also be a strategy to reduce potential competition between age classes^46,47^. The juveniles’ behaviour was less often identified as travelling (fast and straight trajectory) and more often identified as intensively foraging (slow and sinuous trajectory) throughout their entire foraging trip. In contrast, adults foraged most actively at the most distant part of their trip. While developing foraging skills, juveniles can compensate for their low foraging success by increasing their foraging time, as in immature olivaceous cormorants (*Phalacrocorax brasilianus*), which foraged twice as often as adults^48^ or immature royal terns (*Thalasseus maximus*), which have lower diving rates than adults but foraged over longer periods to capture as much prey as the adults^49^. Juvenile RFBs, particularly during the first monitoring period, showed more area-restricted search (ARS) zones per hour than adults. Young birds might be practicing during this portion of the trip, with more attempts to catch prey associated with a high failure rate^50^.

At sea, juveniles foraged in groups during portions of their trips. Half of the tracks of juveniles fitted with GPS loggers included some form of association with other juveniles, sometimes with several individuals foraging together. Because the tracked juveniles represented only a small fraction of the total number of juveniles in the colony, we can conclude for the first time that associations at sea between juvenile RFBs are very common and that they are formed in part opportunistically at sea when foraging opportunities occur. In the vicinity of the island, juveniles may gather in groups for protection, because it decreases the individual probability of harassment by frigatebirds, which use kleptoparasitism as one of their foraging strategies^51^. Juveniles may also gather in groups to use social information from the behaviour of congeners to make decisions^6^. Information transfer is common in the central place foraging of colonial seabirds that exploit unpredictably distributed food patches, such as Guanay cormorants (*Phalacrocorax bougainvillii*), which form a raft at the sea surface that is adjusted to the bearing of returning of cormorants, giving an indication of the location of the food patches^52^. By forming groups before going to sea and during their foraging trips, young RFBs may obtain information on foraging locations from congeners that have better foraging skills or a better knowledge of foraging zones. At sea, associations were more likely to occur during periods of ARS behaviour, suggesting the use of ‘network foraging’^53^, whereby juveniles monitor the movement of other individuals and join those that are intensively foraging (thereby indicating the presence of prey). Miniaturised video recorders also showed that young brown boobies (*Sula leucogaster*) voluntarily followed other conspecifics, which may directly enhance their foraging success and improve foraging and flying skills during their developmental stage^54^. Groups of juvenile RFBs were often observed leaving the island and returning without adults in the vicinity. However, juveniles could randomly follow experienced adults as a strategy to learn and find profitable areas. Indeed, the foraging sessions of juveniles can be more efficient in the presence of adults than in the absence of adults^55^. For example, young captive ringdoves appeared to acquire social information from whatever individuals present, whether they were kin, unrelated conspecifics or heterospecifics. Interestingly, while some passerine birds appear to learn from their parents^55,56^, juvenile RFBs did not associate with their parent voluntarily. Overall, only two associations between a juvenile and any adult were observed. However, this result was probably driven by the low number of adults sampled. Further studies are required to better assess the frequency of juvenile-adult associations.

Juvenile RFBs monitored during the entire post-fledging period increased the duration of their foraging trips and the total time per day they spend foraging with age^20^. In our study, different individuals were fitted with GPS loggers during the 3 successive periods (covering one month in total), reflecting the changes over time since their first flights. Trip duration did not increase between the 3 successive periods. However, the total distance covered and the maximum range significantly increased over time. Five of the 6 individuals tracked between 13 and 29 consecutive days showed a progressive increase in the maximum distance reached each day. These individuals increased their range by more than two-fold between the first day and the last day of tracking, reflecting either a rapid improvement in flying ability, a growing motivation to forage further, or both. An opportunistic visual observation in June 2014 (3.5 months later) reported juveniles present 150 km away from the colony, more than twice the maximum range recorded during the GPS tracking period (≈60 km). Acquiring flying skills appears to be a long process for some raptors^57,58^ and seabirds^27,32,45,59^. Wandering albatrosses progressively increased their daily flight distances to attain adult flight efficiency within their first 6 months at sea^45^. Young brown boobies (*Sula leucogaster*) increased the proportion of time spent gliding during flight with the number of days since fledging, indicating that this energy-saving skill required some time to acquire^32^. Moreover, they were sensitive to strong wind conditions, leading to poor flight stability and potentially reduced prey detection^59^. In contrast, juveniles of Scopoli’s shearwater (*Calonectris diomedea*) were able to fly long daily distances soon after fledging, but as a migratory movement^60^. However, their migratory behaviour reflected less-developed navigational skills compared with adults, as observed in terrestrial birds^61^.

As they age, improved foraging ability coupled with a growing need for food due to a decrease in provisioning by parents could push juvenile RFBs to begin trips earlier in the day and travel further from the colony. The departure time tended to occur earlier in the morning over time. The late departure of juvenile groups during the first period can be explained by the time needed to form large groups. Moreover, the ability to travel longer distances may influence juveniles to leave earlier over time. However, as long as they continued to be fed by their parents, their trips were still constrained by the need to return early to the colony. Over time, juveniles transitioning to independence were observed leaving the colony alone more frequently rather than leaving in groups. Like RFBs, juvenile brown boobies gradually improved their foraging skills during the post-fledging period^21,25,32^. In blue-footed boobies (*Sula nebouxii*), juveniles acquired advanced diving skills soon after their first flight but remained dependent on feeding by their parents for several additional weeks, allowing them to increase their other foraging capabilities^17^.

The independence of juveniles can be progressively induced by parents through a decrease in parental feeding at the end of the post-fledging period^20,57^ or by juveniles that stop begging their parents when foraging for themselves becomes sufficiently profitable^62,63^. Here, feeding frequency and the presence on the nest gradually decreased over time. Juvenile RFBs became independent between the 2 visual surveys conducted at 80 days and 155 days after fledging, which is consistent with previous estimations^20,34,35^. Nazca boobies (*Sula granti*) appeared to use the absence of both parents as part of the decision to depart permanently from the nest^64^. For blue-footed boobies, begging frequency and presence on the nest decreased when the juveniles began to catch more fish^17^. They continued begging for food from their parents 15 days after their first flight, even if they were capable of plunging almost as deep as the adults (more than 3.5 m deep)^17^. The RFB is a shallow diver (maximum of 2.4 m deep)^36^ and thus juveniles may quickly reach depths similar to adults. The beak size was significantly different between the juveniles and the adults. However, no difference was observed between the juveniles from the two successive deployment periods. Since Guo *et al*. found no significant difference in weight, beak size and wing length between the time at fledging and the time at independence^20^, we can hypothesize that the beak may grows mostly after the independence (between the immature and the adult stage). Therefore, the extended transition period does not appear related to morphological limitations.

Tropical sulids are, along with frigatebirds, exceptions among seabirds by displaying a particularly long post-fledging care period, with juveniles becoming gradually independent from their parents^17,27,32^. Both groups feed on similar prey (flying fishes and flying squids) that probably require the acquisition of complex foraging skills. Beyond the difficulty of capturing prey, differences in parental care may be linked to the seasonal abundance of food^26,29^. Indeed, immature northern gannets (*Morus bassanus*), which breed in seasonally productive temperate waters, disperse widely at sea without a post-fledging period of parental provisioning^24^. In tropical regions where resources are known to be scarce, heterogeneous and less predictable than in regions of cold or temperate waters^39,65,66^, an extended parental care period may maximise the fledgling’s survival. The long transition phase observed in typical tropical seabird species such as boobies and frigatebirds^40^ may thus have evolved to allow a long period of learning, which is necessary in an unpredictable environment.

## Materials and Methods

### Study site and period

The data were collected on Europa Island (22.3°S, 40.3°E; local time = GMT+3), which is located in the Mozambique Channel, 300 km from the coast of Madagascar and 500 km from the mainland coast of Africa. Europa hosts 2 800-3 800 pairs of breeding RFB, all located in the dry *Euphorbia stenoclada* forest on the northern part of the island^51^. GPS tracking and visual surveys were carried out between 27 January and 4 March 2014, with 2 main periods of deployment (25 Jan-2 Feb and 15 Feb-17 Feb). All juveniles studied were recently fledged and within the first 2 weeks after their first flight.

Opportunistic observations are also reported from later fieldwork in June 2014 and November 2014. In addition, to determine when juveniles became totally independent from the parents, 3 nests were monitored by camera trapping (see below). One nest was monitored in 2014 (18 Feb-2 Mar) and two nests were monitored in 2015 (20 Feb-7 Mar and 9 Mar-1 Apr), corresponding to the periods +15 days, +60 days and +80 days from fledging, respectively.

### GPS tracking and associations

Adults and juveniles were fitted with 20 g (32 × 22 mm) IGotU GPS loggers (Mobile Action Technology) that recorded their position every 2 min (adults) or 1 min (juveniles). GPS loggers were waterproofed with a PVC sheath and attached under the three central tail feathers using Tesa tape^67^. Birds were chosen randomly and captured by hand or with a 6-m telescopic fishing pole fitted with a nylon noose (for birds nesting higher in the trees). A labile dye was used to mark birds on the tail or the breast to identify them rapidly from a distance. Birds were measured (culmen height and length, wing length) at the time of recovery of the GPS logger. A total of 380 tracks were collected from 7 adults and 34 juveniles, including 3 cases where the juvenile and one of its parents were both tracked. For the analysis, 3 consecutive periods of 12 days were defined: (1) 27 Jan-7 Feb, (2) 8 Feb-19 Feb and (3) 20 Feb-4 Mar, hereafter referred to as P1, P2 and P3, respectively. Depending on the GPS loggers, the tracking period of each individual covered one, two or all three periods. Six juveniles were tracked over 13 to 29 consecutive days. Five outliers emerging from the distribution of the trip durations were removed for all analyses to describe the typical behaviour of the species, including 3 tracks recorded during a cyclone (Guito) that crossed the Mozambique Channel between 18 and 22 Feb 2014. Three incomplete tracks where the loggers failed to record the entire trip were also removed. The duration of the foraging trip (h), total distance covered (km) and the maximum range from the colony (km) were calculated for each track. Tracks were plotted on maps generated with the R package *ggmap* ^68^ or over bathymetric maps obtained from the one arc-minute resolution GEBCO bathymetric dataset using the R package *marmap* ^69^.

To study the associations between individuals during foraging trips, we made pairwise comparisons between all locations from one bird with all the locations from the other birds. To our knowledge, no similar analysis has been conducted in the literature to identify association events from the GPS tracking of a seabird. After testing several values of time difference and distance parameters, association events were identified when the differences in latitude and longitude for two individuals were both lower than 0.002° (c. 280 m) within a time interval of 30 s. All associations were carefully checked visually. Associations identified within 50 m of the nest were not taken into account to limit the study of associations to those occurring at sea. To avoid very short random encounters, tracks containing only one association event were ignored.

### Behaviour labelling and ARS

To determine the different behaviours of individuals during their foraging trips, we used the Expectation Maximisation binary Clustering (EMbC) algorithm^70^, a variant of the Expectation–maximisation algorithm in Maximum Likelihood Estimation of Gaussian Mixture Models. The EMbC algorithm is a robust, non-supervised multi-variate clustering algorithm leading to meaningful local labelling of each GPS location that can be easily linked to biological interpretations. The clustering is based on two variables: the speed and the turning angle obtained from successive locations. Initially, all tracks were linearly interpolated with one location every 2 minutes, and the maximum speed was set to 90 km. h^−1^ ^36^. Each GPS location was labelled with one of 4 behaviours delimited by intervals of speed and turning angle assigned by the EMbC algorithm: resting (0 to 6 km.h^−1^ and 0 to 0.30 radians), intense foraging (0 to 14 km.h^−1^ and 0.3 to 3.14 radians), travelling (6 to 90 km.h^−1^ and 0 to 0.43 radians), and relocating (14 to 90 km.h^−1^ and 0.43 to 3.14 radians). For more details see Mendez *et al*.^71^. To compare the behaviour of adults and juveniles, trips from both age classes were treated simultaneously in the analysis conducted with the *EMbC* R-package. All analyses were conducted in R 3.2.3^66^.

Zones of area-restricted searching (ARS) were defined when at least 3 successive locations were labelled intensive foraging by the EMbC algorithm^33^. To simplify the description of the different behaviours along the trajectory, we merged ARSs when less than 4 locations labelled with another behaviour were observed between them^33^. The number of ARSs per hour, their duration and their maximum range were calculated. Each ARS was summarised for one central location by taking the median latitude and longitude.

Kernel estimation^67^ was used to determine the utilisation distribution (UD) probability based on ARS zones. Kernel density estimates offer the advantage of being widely used to identify population-level core habitat areas. We used the function *kernelUD* implemented in the R package *adehabitatHR* ^68^ using the reference bandwidth, which produces contiguous cores without over-smoothing, choosing a secant projection and a narrow zone to minimise the distortions in a map generated from projection. To estimate the size of the general (95%) and core (50%) foraging areas, we used the function *getverticeshr* with an adapted local projection (Europa: Moznet / UTM zone 37 S).

### Visual surveys

Visual surveys lasting between 30 min and 3 h were carried out at sunrise to study birds’ departure at sea (n = 14 days) and before sunset when birds returned to the colony (n = 6 days). All observations were made in the field of vision (approximately 500 m wide) looking in the direction of the sea from a fixed elevated point (10 m) on a dune located 20 m away from the shore and between 0.5 and 1 km from the colony. Counting occurred from a line corresponding approximately to the beachfront. Each bird or bird group that crossed this line was counted and identified as adult or juvenile, which is easily distinguishable by plumage and beak colour. Bird groups headed in the same direction were usually composed of individuals following each other within 5 metres (and never more than 15–20 metres). Separate groups were often spaced tens to hundreds of metres apart and thus were readily distinguishable.

### Camera trapping and feeding frequency

Scoutguard SG 550 cameras were installed near active nests. At each movement, the trigger was activated to take a picture every 5 sec. All feeding events occurring in the field of the camera were recorded. The framing was chosen according to the preferential roost of the juvenile, recognisable by the pile of droppings lying below. There were no visual observations that indicated feeding events outside the nest, but the feeding frequency could be underestimated if feeding occurred outside the frame. Standardised tours checking a group of 20–25 nests were conducted throughout the study period at 15, 60, 80 and 155 days after fledging. Standardised tours were made after sunset (between 21:00 and 23:00) or before sunrise (between 5:00 and 6:00) to determine the presence or absence of juveniles and adults on the nest during the night. Combined with the camera trapping on some nests, the tours allowed the linking of the presence on the nest to the feeding frequency.

### Statistical analysis

As some individuals were tracked during several successive trips, linear mixed-effects models with ‘individual’ as a random factor were applied to avoid pseudoreplication. We used the function *lmer* from the R package *lme4* ^69^ to test for differences in trip parameters between study periods and age classes. Tukey’s HSD test was used to calculate post hoc comparisons on each factor in the model using the function *glht* from the R package *multcomp* ^70^. When the residuals were not normally distributed, variables were log- or square-root transformed. When the data still did not meet the assumptions, we used non-parametric tests with a Kruskal-Wallis rank sum test followed by a Tukey and Kramer (Nemenyi) test for pairwise comparisons with a Tukey-Dist approximation for independent samples from the R package *PMCMR* ^71^. Values of the dependent variables are given as the mean ± standard deviation. Correlations were made using Pearson’s coefficient. The Marascuilo procedure^72^ was used to compare the pairwise proportions of the behaviours defined with the EMbC algorithm^70,73–79^ among study periods and age classes. A Chi-square test was used to evaluate the link between associations and ARS.

### Ethics Statement

The deployment of GPS on birds lasted less than 10 minutes and no birds left the nest as a result of handling. The field procedures were approved by the Préfet des Terres Australes et Antarctiques Françaises. This work was part of the programme EARLYLIFE, funded by a European Research Council Advanced Grant under the European Community’s Seven Framework Program FP7/2007e2013 (Grant Agreement ERC-2012-ADG_20120314 to Henri Weimerskirch). All methods were performed in accordance with the relevant guidelines and regulations.

### Data availability

Data will be made accessible under the Dryad Platform (http://www.datadryad.org/).

## Electronic supplementary material

Supplementary Information
