# Supplementary material for: Ontogeny of foraging behaviour in juvenile red-footed boobies (Sula sula)
Source: Sci Rep. 2017 Oct 24;7:13886. doi: 10.1038/s41598-017-14478-7 (PMC5654766; doi:10.1038/s41598-017-14478-7)
Supplement: Supplementary file 1 — Supplementary Information [file 41598_2017_14478_MOESM1_ESM.pdf]

Ontogeny of foraging behaviour in juvenile red-footed boobies (*Sula sula*)

Loriane Mendez[1*], Aurélien Prudor[1], Henri Weimerskirch[1]

[1]Centre d'Etudes Biologiques de Chizé (CEBC), UMR7372 CNRS, Université de La Rochelle, 79360 Villiers-en-Bois, France

*corresponding author: loriane.mendez@cebc.cnrs.fr

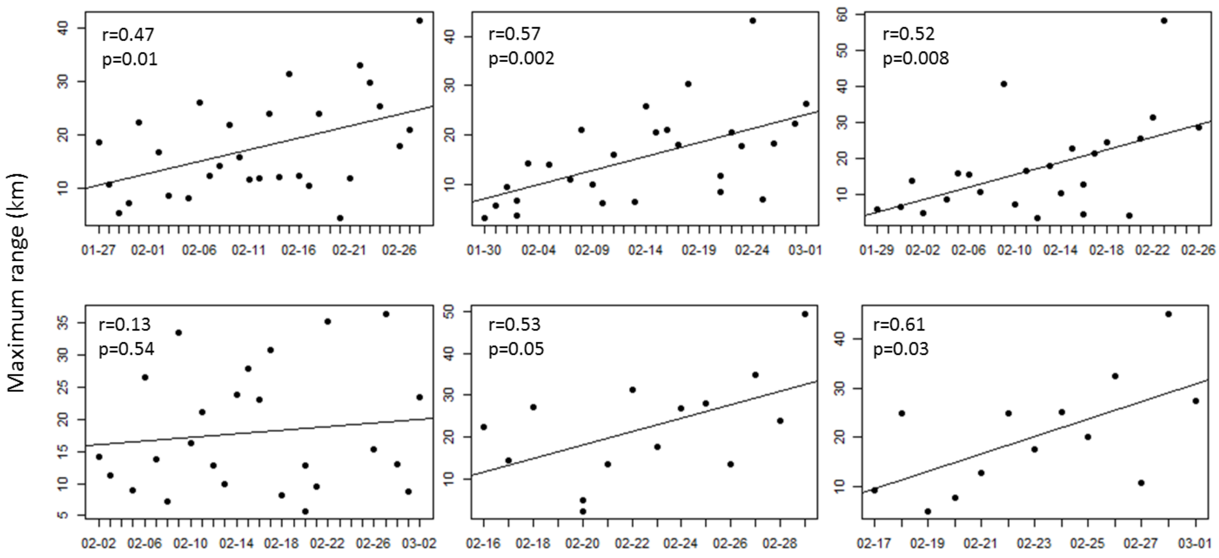

Supplementary Figure S1. Maximum range (km) as a function of time for 6 juveniles tracked during 13 to 29 consecutive days (r: Pearson's correlation coefficient, p: p-value of the correlation test). The continuous line represents the linear regression. Dates are given as dd-mm.

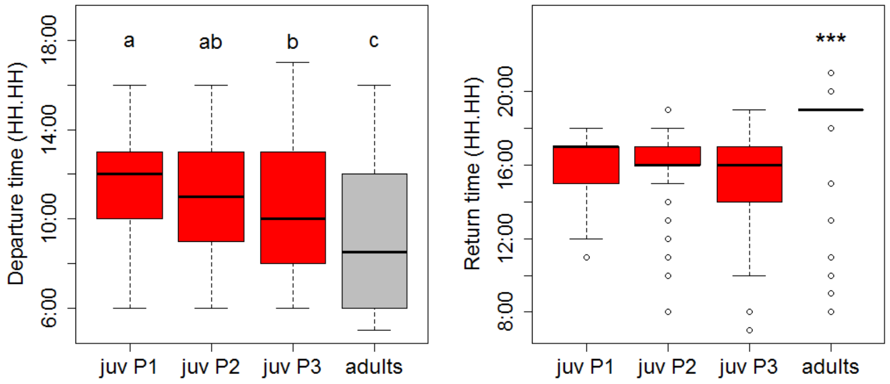

Supplementary Figure S2. Departure and return time of juvenile (red) and of adult (grey) red-footed boobies during the 3 consecutive monitoring periods (P1, P2, P3). Different letters above boxes indicate significant differences (Tukey's HSD test).

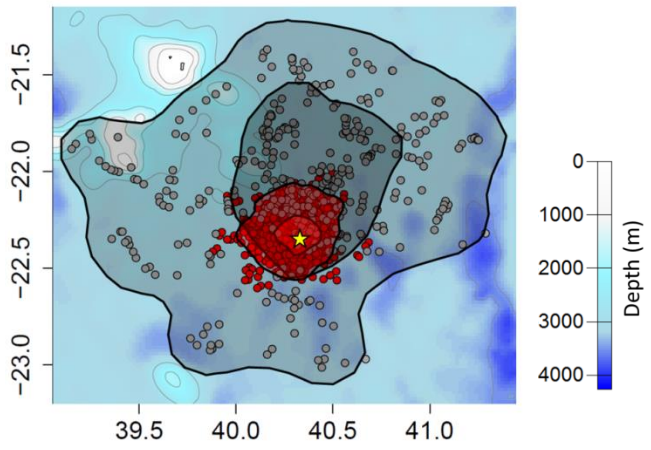

Supplementary Figure S3. General (95% kernel density estimation, light shading) and core (50% kernel density estimation, dark shading) distribution of area-restricted search (ARS) zones in juvenile (red) and adults (grey) red-footed boobies from Europa (indicated by a yellow star) superimposed on bathymetric map generated with the R package *marmap* using the software R version 3.2.3 (2015-12-10, www.R-project.org).

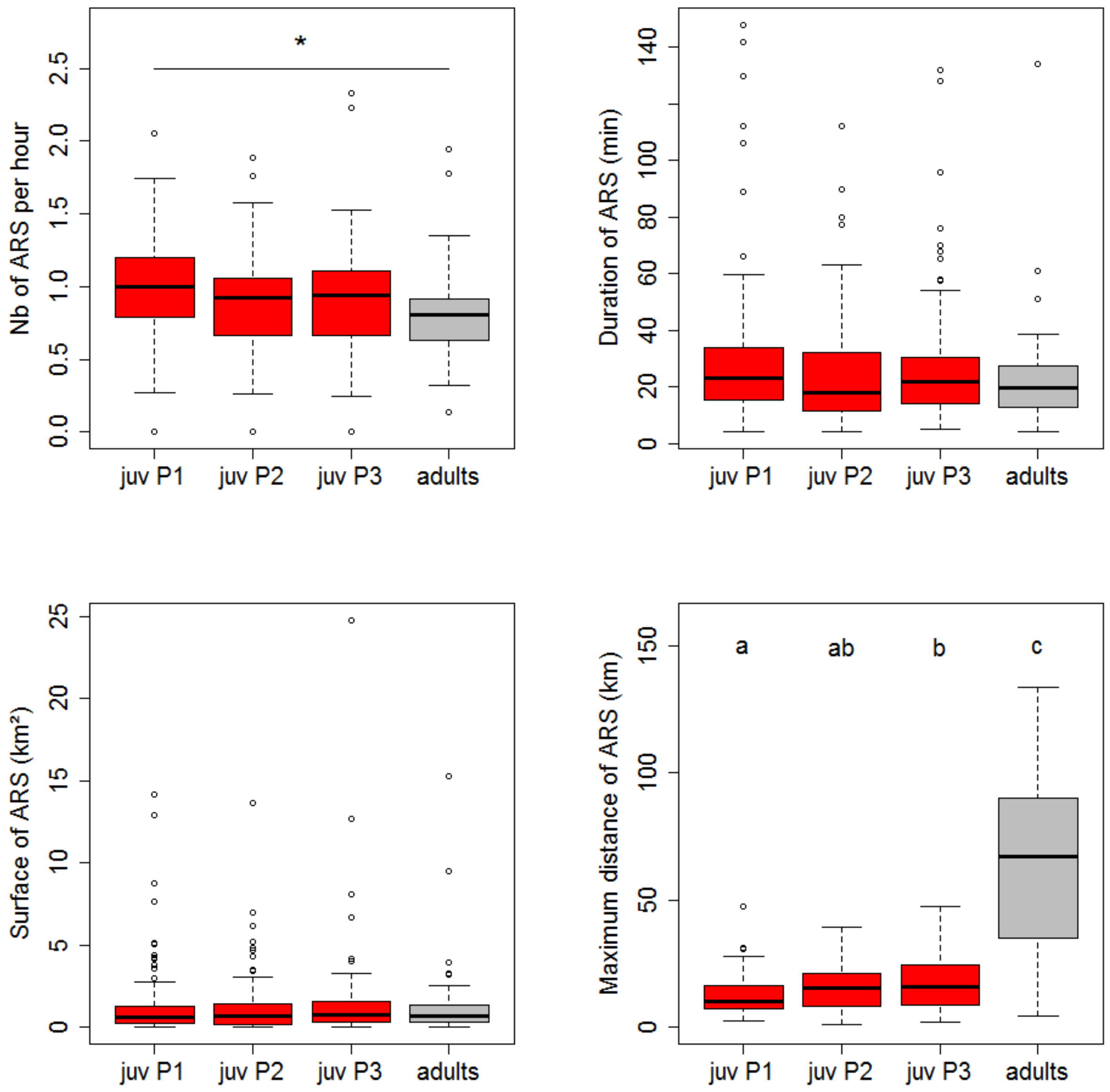

Supplementary Figure S4. Number per hour, duration (min), surface (km^2) and maximum distance from the colony (km) of area-restricted search (ARS) zones in juvenile (red) and adult (grey) red-footed boobies during the 3 consecutive monitoring periods (P1, P2, P3). Different letters above boxes indicate significant differences (Tukey's HSD test).

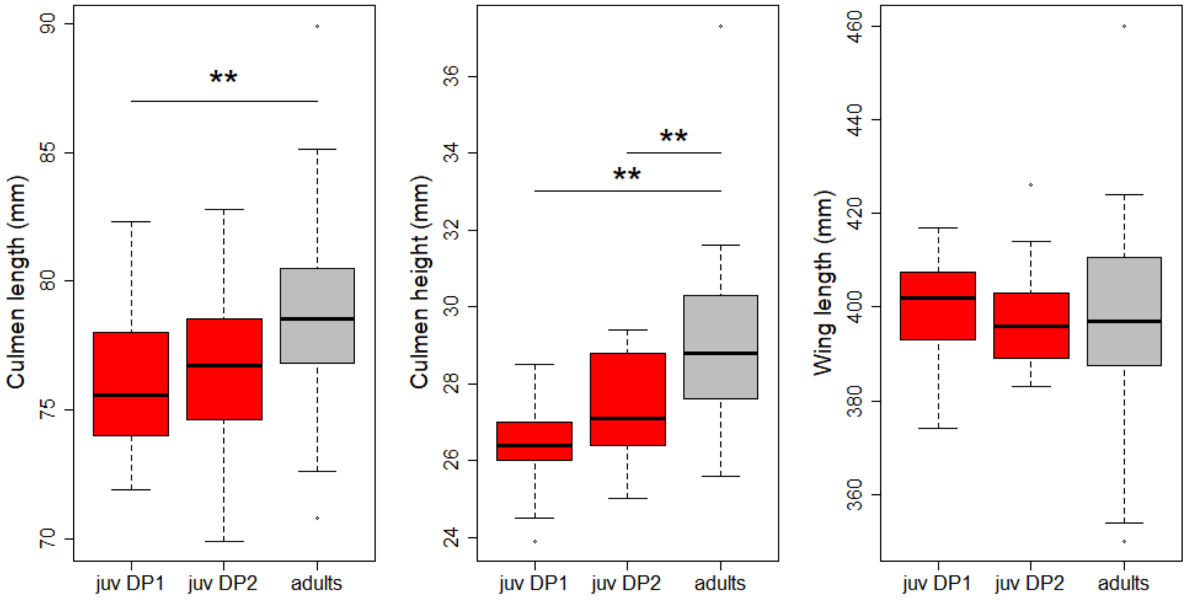

Supplementary Figure S5. Culmen length (mm), culmen height (mm) and wing length (mm) of juvenile (red) and adult (grey) red-footed boobies according to the deployment period of the GPS loggers (DP1: 25 Jan-2 Feb 2014, DP2: 15 Feb-17 Feb 2014).
